# Pre-marital sexual debut and its associated factors among in-school adolescents in eastern Ethiopia

**DOI:** 10.1186/1471-2458-12-375

**Published:** 2012-05-24

**Authors:** Lemessa Oljira, Yemane Berhane, Alemayehu Worku

**Affiliations:** 1 College of Health Sciences Haramaya University, Harar, Ethiopia; 2 Addis Continental Institute of Public Health, Addis Ababa, Ethiopia; 3 School of Public Health, Addis Ababa University, Addis Ababa, Ethiopia

**Keywords:** Ins-school, Adolescents, Pre-marital, Sexual debut

## Abstract

**Background:**

More adolescents in Ethiopia are in school today than ever, but few studies have assessed the sexual behaviour of these learners. Thus, this study tried to assess pre-marital sexual debut and factors associated with it among in-school adolescents in Eastern Ethiopia.

**Methods:**

A cross-sectional school-based study was conducted using a facilitator guided selfadministered questionnaire. Respondents were students attending regular school classes in fourteen high schools. The proportion of adolescents involved in pre-marital sexual debut and the mean age at sexual debut was computed. Factors associated with pre-marital sexual debut were assessed using bivariate and multivariable logistic regression.

**Results:**

About one in four, 686 (24.8%) never married in-school adolescent respondents reported pre-marital sexual debut of these 28.8% were males and 14.7% were females (*p* < 0.001). Pre-marital sexual debut was more common among adolescents who had their parents in urban areas (Adjusted OR and [95% CI] =1.42 [1.17–1.73]), who received higher pocket money per month (Adjusted OR and [95% CI] = 1.56 [1.19–2.04]), who perceived low self-educational rank (Adjusted OR and [95% CI] =1.89 [1.07–3.34]) and who lived in rented houses (Adjusted OR and [95% CI] =1.32 [1.03–1.70]). The females and those who were less influenced by external pressure were more protected against pre-marital sexual debut (Adjusted OR and [95% CI] = 0.44 [0.35–0.56; 0.62 [0.52–0.74, respectively]) than their counterparts.

**Conclusion:**

A significant proportion of in-school adolescents were engaged in sexual relationship. Thus, public health interventions should consider the broader determinants of premarital sexual debut, including the ecological factors in which the behavior occurs.

## Background

In Ethiopia, an increasingly large number of adolescents are enrolled in high schools and they often live away from parental guidance. Early initiation to sexual intercourse without having proper protection has been one of the concerns [1]. Previous studies in Ethiopia indicated that from 17.8% to 21.5% of the adolescents are sexually active [2,3] and only about 40% of the sexually active adolescents reported consistent use of condom [2,4,5]. Studies elsewhere have also documented early sexual initiators were more likely to report undesired consequences of sexual initiation such as teenage motherhood, not using condom at first sex and sexually transmitted infections [6]. Adolescents are also likely to have a sexual partner who is five or more years older and be involved in multiple sexual partnerships [7].

Studies conducted in Ethiopia identified inconsistent predictors of sexual debut. Gender was associated with both increased and decreased pre-marital sexual debut. Reading or viewing sexually explicit materials, alcohol drinking, khat chewing, and ever having a boy or a girlfriend were associated with increased pre-marital sexual debut, while living with parents was associated with decreased pre-marital sexual debut. Similarly, the median age at sexual debut was also a point of controversy; some studies report that median age at sexual debut, particularly for females, overlap with that of marriage and some studies also failed to differentiate whether sexual debut took place before marriage or within marital relationships [2,3,5,8]. Studies in other countries also revealed that male gender, alcohol consumption, substance use and living out of parental close supervision were associated with sexual debut [9-13].

Even though the number of school going adolescents is continuously mounting in Ethiopia, studies rarely assessed adequately their sexual behavior and the context in which the behavior occurs; the studies were done on limited samples of students and schools. [2,3,5]. Thus, this study tried to assess pre-marital sexual debut and the factors associated with it among in-school adolescents in Eastern Ethiopia.

## Methods

The study design was a cross-sectional school based survey with internal comparison. The study was conducted in Eastern Ethiopia, and it involved fourteen randomly selected schools found in fourteen different districts. The sample size studied (N = 2880) was primarily determined for baseline study of a cluster randomized educational intervention study. All the students attending regular classes in the selected high schools were eligible for the study and the respondents were randomly selected by considering male-female proportion (72% male and 28% females) based on enrollment data for that academic year. Data were collected by a facilitator guided self-administered structured questionnaire adapted from sexual and reproductive health questionnaires of WHO [14]. Data were collected from all students selected from one school simultaneously to overcome information contamination. The data collection was facilitated by gender-matched facilitators. Facilitators were selected from faculty members at Haramaya University. A minimum of two facilitators who can speak the local language facilitated the data collection process per classroom. The questionnaires were prepared in English, and a process of translation and back translation, with a comparison by independent bilingual language experts ensured Consistency.

The dependent variable was pre-marital sexual debut and it refers to heterosexual intercourse (putting penis inside vagina before marriage), while the independent variables were gender, area of residence, father’s educational status, mother’s educational status, birth order, perceived self- educational rank, family wealth index, pocket money per month, living arrangement during high school education, and perceived self-sufficiency. Perceived self-efficacy was measured by how sure respondents were to perform or refuse to perform the specified activity. For example a respondent was asked how confident s/he had been to refuse sex when s/he was not ready for it with someone under different circumstances on scale ranging from 1 (not at all sure) to 5 (very sure). The scores were summed and the overall mean score was computed, and finally the respondent who scored equal to or above the mean score of twenty self-efficacy questions were taken as self-confident and below as not self-confident.

Data were double-entered onto the EPI-data Version 3.1 software by defining legal values for each variable and setting skip patterns. The double-entered data were validated and exported to Stata/SE 11.0 soft-ware. The proportion of pre-marital sexual debut and the mean age at sexual debut were computed and compared for male and female. Factors associated with pre-marital sexual debut at bivariate were identified and the variables with p-value of 0.25 and less were taken to multivariable analysis and the model was built with backward elimination. Finally, the p-values less than 0.05 were considered statistically significant.

The study was conducted after securing approval of Institutional Review Committee of Haramaya University and the necessary permission from other concerned educational authorities. The confidentiality of information was maintained by excluding personal identifiers and data were collected after securing informed consent and/or assent from every respondent.

## Results

Of the 2880 students requested to fill out the self-administered questionnaire, 2766 students responded, making the response rate 96%. More than two-third, 1985 (71.8%) of the respondents were male. The age of the respondents ranged from 14 to 19 years and the mean age was 17.1 (1.2) (Table 1). About one in four, 686 (24.8%) never married in-school adolescents reported premarital sexual debut; significantly more males (28.8%) than females (14.7%) reported premarital sexual debut (*p* < 0.001). The age at pre-marital sexual debut ranged from 13 to 19 years, and the mean was 15.6 (1.7). Males had lower (15.5 years) mean age at pre-marital sexual debut compared to females (16.0 years) (*p* = 0.03).

**Table 1:** Background characteristics of in-school adolescents, Eastern Hararge Zone, Oromia Regional State, Eastern Ethiopia, 2011

**Variables**	**Category**	**Number**	**Percentage**
**Respondent’s sex:**	Male	1985	71.8
	Female	781	28.2
**Age group:**	14–15	286	10.3
	16–71	1224	44.3
	18–19	1256	45.4
**Respondent’s Ethnicity:**	Oromo	2461	89.0
	Amhara	221	8.0
	Other	84	3.0
**Respondents religion:**	Islam	2342	84.7
	Christian	353	12.8
	Other	71	2.5
**Respondent’s birth order:**	Middle	1950	70.5
	First	715	25.9
	Only child	101	3.6
**Perceived self-educational rank:**	Top	925	33.4
	Average	1780	64.4
	Weak	61	2.2
**Living arrangement for education purpose:**	Family/relative	1799	65.0
	Rental alone	393	14.2
	Rental in group	574	20.8
**Pocket money from family per month (in Birr)**	< 100	1324	39.8
	100–200	1100	47.9
	>200	342	12.3

### Predictors of pre-marital sexual debut

The logistic regression found out that the in-school adolescents whose family residential area was from urban area were more likely to engage in pre-marital sex than those from rural area (Adjusted OR and [95% CI] =1.42 [1.17 - 1.73]). Females were 56% less likely to report pre- marital sexual debut compared to males (Adjusted OR and [95% CI] = 0.44 [0.35–0.56]). As the amount of pocket money per month increased, the pre-marital sexual debut also increased. Compared to the in-school adolescents who got less than 100 Ethiopian Birr per month, those who got 100 to 200 or more than 200 per month were more likely to report pre-marital sexual debut in an increasing order (Adjusted OR and [95% CI] = 1.28 [1.05–1.55]), and 1.56 [1.19–2.04]), respectively). Respondents who perceived themselves to have low self-educational rank were almost two-times more likely to engage in pre-marital sexual intercourse compared to those who perceived themselves to have top rank (Adjusted OR and [95% CI] =1.89 [1.07–3.34]). The respondents who were living alone during high school education were more likely to report pre-marital sexual debut, compared to living with parents/relatives (Adjusted OR and [95% CI] =1.32 [1.03–1.70]). Adolescents who perceived themselves to be self-sufficient in “*saying no”* to external pressure to have sexual intercourse when they felt not ready were 38% less likely to report pre-marital sexual debut, compared to their counterparts (Adjusted OR and [95% CI] =0.62 [0.52–0.74]) (Table 2).

**Table 2:** Logistic regression indicating factors associated with pre-marital sexual debut among never married in-school adolescents Eastern Hararge Zone, Oromia Regional State, Eastern Ethiopia 2011

Variable	Pre-marital sexual debut		Crude OR (95% CI)	Adjusted OR (95% CI)
	Yes	No		
Family Residence:				
Rural	444	1457	1	1
Urban	242	623	1.27 (1.06–1.53)	1.42 (1.17–1.73)
Respondent’s sex:				
Male	571	1414	1	1
Female	115	666	0.43 (0.34–0.53)	0.44 (0.35–0.56)
Family Wealth Index:				
Low	142	449	1	
Average	436	1294	1.07 (0.07–1.32)	1.19 (0.93–1.51)
High	108	337	1.01 (0.76–1.35)	1.04 (0.75–1.44)
Pocket money (Birr):				
< 100	277	1047	1	1
100–200	296	804	1.39 (1.15–1.68)	1.28 (1.05–1.55)
>200	113	229	1.87 (1.44–2.42)	1.56 (1.19–2.04)
Mother’s educational status:				
No education	441	1366	1	1
Primary	136	409	1.03 (0.83–1.29)	1.04 (0.82–1.31)
Secondary	38	89	1.32 (0.89–1.96)	1.30 (0.86–1.97)
12 and above	16	30	1.65 (0.89–3.10)	1.74 (0.91–3.33)
Respondent’s birth order:				
Middle	490	1460	1	1
First	163	552	0.88 (0.72–1.08)	0.87 (0.70–1.08)
Only child	33	68	1.45 (0.94–2.22)	1.30 (0.81–2.08)
Perceived self-educational rank:				
Top	214	711	1	1
Average	415	1329	1.13 (0.94–1.36)	1.19 (0.98–1.44)
Low	21	40	1.74 (1.01–3.02)	1.89 (1.07–3.34)
Living arrangement:				
With family/relative	437	1362	1	1
Rental alone	116	277	131 (1.03–1.66)	1.32 (1.03–1.70)
Rental in group	133	441	0.94 (0.75–1.17)	0.91 (0.72–1.14)
Perceived self-confidence:				
Not confident	389	904	1	1
Confident	297	1176	0.59 (0.49–0.70)	0.62 (0.52–0.74)

## Discussion

Six hundred eighty six (24.8%) of the never married respondents had a penetrative pre-marital sexual debut and 79.0% of them had initiated sex before 18 years of age. Males initiated sex at earlier age compared to the females. The mean age at sexual debut for females was roughly comparable with lower median age reported from other countries [12,15] and lower than the earlier Ethiopian DHS report, and the mean age at sexual debut for males in the current study was lower than the previous estimates [8,16-18]. This is may be because of the difference in the study population; as this study involved only in-school adolescents within narrow age range (13 - 19 years) while the DHS covered a wider area and diverse population. The median age at sexual debut for males was lower than that for females in this study. This contradicts with the previous DHS reports. This is may be due to the difference in the study population. This study dealt with only unmarried in-school adolescents while the DHS studied a wider age range and different population groups. Moreover, the difference could also be attributed to difference in the period of the study, which may suggest a changing trend in easiness of reporting sexual matters and increase in pre-marital sexual debut coupled with a rising age at marriage.

Family residential area was associated with pre-marital sexual debut. The adolescents from urban families were more likely to engage in pre-marital sex than those from rural area. This finding is consistent with the study conducted in South Africa, which identified periurban residence association with earlier age at first sex [17]. This may be due to the more liberal life styles in urban areas compared to cultural conservatism in rural areas, and may also be attributed to easiness of reporting pre-marital sexual debut by urban adolescents.

Pre-marital sexual debut was associated with gender, in that females were 56% less likely to report pre-marital sexual debut compared to males. Similar findings were documented from other studies [12]. The possible explanation for such differences can be due to a double standard cultural expectations of virginity at marriage for females while lesser cultural pressure on males or even greater tolerance towards male pre-marital sexual experimentation.

Family wealth index was not associated with pre-marital sexual debut; however, the amount of pocket money per month was a strong predictor, in that the higher the amount of pocket money per month, the greater was the pre-marital sexual debut. This is may be due to lack of skills to wisely use the pocket money which may be above what was required for subsistence. Particularly as the association was significant for male gender may be because more pocket money may predispose to commercial sex (Adjusted OR and [95% CI] = 1.29 [1.04–1.60], 1.50 [1.12–2.02]; and 1.18 [0.75–1.85], 1.84[0.92–3.66] for males and females corresponding to the pocket money per month respectively).

Perception of self-educational rank was associated with pre-marital sexual debut, in that those who perceived to have lower educational rank were almost two times more likely to engage in pre-marital sexual debut, compared to those who perceived top educational rank. This may be because low educational rank perceivers may easily be involved in a “*group study”* practiced commonly by in-school adolescents in the study area which may easily expose them to sexual experimentation.

Rental alone living arrangement during high school education was associated with increased pre- marital sexual debut compared to living with parents/relatives; this finding is consistent with other studies [6,12]. This may be due to the absence of parental monitoring and guidance.

Perceived self-confidence to refuse sexual intercourse when not ready for it was found to be protective from engaging in pre-marital sexual debut. Only 53.2% of in-school adolescents reported having such confidence and adolescents who had perceived self-confidence were 38% less likely to engage in pre-marital sexual debut. This may be because adolescents who perceived less self-confidence may easily be influenced by peer or adults’ pressure to engage in sexual activity that they may regret about it afterwards. On the contrary, students with more self- confidence may have a higher sense of future achievement and feel too self-confident to resist external influence to have sex.

This study involved a large sample size with an excellent response rate. The major limitation of this study was its sensitivity as it dealt with the personal lives of in-school adolescents. Even though data were collected by facilitator guided self-administered method and the anonymity of the information was maintained to make respondents establish a sense of trust, it may be difficult to overcome the risk of social desirability bias completely. The fact that the study was limited to in-school adolescents who were found at school during the time of data collection was another limitation as it may be difficult to generalize the result to out of school and drop-outs that might have different life experiences.

## Conclusion

In conclusion, a large proportion of the in-school adolescents initiated pre-marital sex. The factors associated with this were both individual factors (gender, perceived self-educational rank and perceived self-confidence of refusing sexual intercourse if not ready, amount of pocket money per month) and ecological factors (family residential area and living arrangement during high school education). Thus, public health interventions directed to in-school adolescents should address the broader determinants of pre-marital sexual debut including the ecological factors in which the behavior occurs in addition to individual level factors considering diverse nature of these target population groups and maintaining their different choices.

## Competing interests

Authors declare that they have no competing interests

## Authors’ contribution

All authors participated in the design of the study. LO and YB participated in the data collection and follow-up. LO analyzed the data. All authors participated in the drafting and approval of the manuscript.

## Pre-publication history

The pre-publication history for this paper can be accessed here:

http://www.biomedcentral.com/1471-2458/12/375/prepub
